# Apple Watch-guided diagnosis of AVNRT in a pregnant woman—A case report and literature review

**DOI:** 10.3389/fcvm.2022.985421

**Published:** 2022-11-07

**Authors:** Maja Hawryszko, Grzegorz Sławiński, Dariusz Kozłowski, Ewa Lewicka

**Affiliations:** Department of Cardiology and Electrotherapy, Medical University of Gdańsk, Gdańsk, Poland

**Keywords:** AVNRT, pregnancy, Apple Watch, mobile health (mHealth), arrhythmia

## Abstract

Cardiac arrhythmias occurring during pregnancy pose a therapeutic problem as antiarrhythmic drugs can be potentially harmful to the fetus. A 35-years-old woman in the 20^th^ week of pregnancy was admitted to the Department of Cardiology due to the first episode of arrhythmia in her life. During the event, the patient was wearing an Apple Watch Series 6, which records a 30-sec single-channel ECG. The recording showed narrow QRS complex tachycardia of 216 bpm, and short RP interval and atrioventricular nodal reentrant tachycardia (AVNRT) was recognized. Due to the mild nature of the arrhythmia, antiarrhythmic pharmacotherapy was not initiated. The use of mobile health (mHealth) devices such as wearables and health monitoring applications is now a valuable addition to routine cardiac diagnostics for patients of all ages and levels of cardiovascular risk.

## Introduction

Cardiac arrhythmias occurring during pregnancy pose a therapeutic problem as antiarrhythmic drugs can be potentially harmful to the fetus. Therefore, in the presence of mild arrhythmias, antiarrhythmic drug therapy is not recommended. The use of mobile health (mHealth) devices such as wearables and health monitoring applications is now a valuable addition to routine cardiac diagnostics for patients of all ages and levels of cardiovascular risk (1).

## Case description

**Figure 1:**
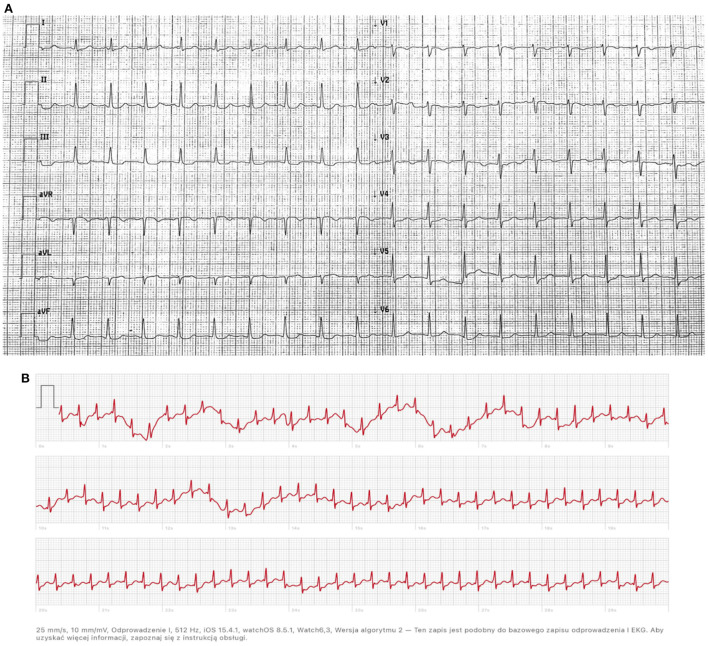
**(A)** ECG on admission to hospital: sinus tachycardia 113 bpm, normal cardiac axis, T-waves inversion in leads V1 - V3; **(B)** Recording from Apple Watch: narrow QRS complex tachycardia of 216 bpm with short RP interval.

A 35-years-old woman in the 20^th^ week of pregnancy was admitted to the Department of Cardiology due to the first episode of arrhythmia in her life. During normal activities, she developed palpitations and pre-syncope symptoms. Until now, she had no concomitant diseases and had no risk factors for cardiovascular disease. She was not taking any medications. On admission, the patient did not report any symptoms, was in good general condition, and was without any abnormalities in the physical examination. ECG showed sinus tachycardia 113 bpm, normal cardiac axis, and T-waves inversion in leads V1—V3 (Figure 1A).

During the event, the patient was wearing an Apple Watch Series 6, which records a 30-sec single-channel ECG. The recording showed narrow QRS complex tachycardia of 216 bpm and short RP interval (Figure 1B), and atrioventricular nodal reentrant tachycardia (AVNRT) was recognized. During hospitalization, no cardiac arrhythmia was found during telemetric ECG monitoring, and transthoracic echocardiography showed no abnormalities. Due to the mild nature of the arrhythmia, antiarrhythmic pharmacotherapy was not initiated. The patient was informed about the possibility of terminating the arrhythmia with the Valsalva maneuver.

## Discussion

Cardiac arrhythmias may appear for the first time in pregnancy, but they may also aggravate or recur previously observed arrhythmias. Atrial fibrillation and paroxysmal supraventricular tachycardia (PSVT) are, in addition to premature beats, the most common forms of arrhythmia in pregnancy. Atrioventricular recurrent tachycardia (AVNRT) predominates among PSVT. It is estimated that approximately 20% of patients with pre-pregnancy supraventricular tachycardia will exacerbate their symptoms during pregnancy.

According to the 2019 guidelines for the management of patients with supraventricular tachycardia, vagal maneuvers are a first-line treatment for AVNRT during pregnancy. It is also recommended to avoid the use of antiarrhythmic drugs in pregnant women with mild symptoms or rare and short episodes of arrhythmia (2). If symptoms are present and the arrhythmia is not tolerated by the woman, and if periodic disturbances in uteroplacental flow are present, treatment with a cardioselective beta-blocker should be considered, preferably after the first trimester of pregnancy. If there is no improvement after the treatment, the substrate of the arrhythmia may be ablated, preferably after the end of pregnancy (3).

Recently, watches have turned into devices that not only show the time, but provide a lot of information about life activity, sleep, and its phases, and some of them also have the function of recording a real-time electrocardiogram (ECG). By enabling patients to take their ECG recordings in situations such as palpitations or presyncopes, clinicians can increasingly review hard-to-reach arrhythmias and those occurring less often thanks to registration using a smartwatch (4). Research has been carried out on the diagnostic accuracy of smartwatches in all cardiac arrhythmias. They show that the detection of cardiac arrhythmias with smartwatches available on the market is possible with very high diagnostic accuracy. The overall sensitivity, specificity, and accuracy of these digital systems were 100, 95, and 97%, respectively. PPV and NPV were 85 and 100%, respectively (5).

In the case described above, the patient used the smartwatch ECG function when she felt palpitations. However, not all patients experience episodes of arrhythmia to benefit from the ECG recording. A good example is an atrial fibrillation, which is often asymptomatic. Measuring the heart rate, but also detecting cardiac arrhythmias basically uses two different technologies: photoplethysmography (PPG) and electrocardiography. The resulting pulse wave sequences can be analyzed with the help of special algorithms (their regularity and irregularity), and arrhythmias can be detected using the variability of the pulse frequency. This information is analyzed by numerous available applications designed for this purpose (6). A number of studies have demonstrated the high accuracy of a wide variety of mobile AF detection devices. However, studies are still being conducted to confirm such large-scale use of patients (7). One of the largest studies is conducted by Perez et al. (8), where of the 450 participants who were monitored with ECG patches, 153 identified AF—resulting in a diagnostic AF efficiency of 34%. In subjects 65 years of age or older, AF was detected in 35% of patients, while among participants under 40 years of age, the diagnostic efficiency of AF was 18%. The goal of the Apple Heart Study was to evaluate the ability of the Irregular Heart Rate Alert algorithm to identify AF with the Apple Watch application by consumers. Among participants who received a report of an abnormal heart rate, 84% of reports were consistent with AF (8).

Nowadays, when health awareness and the need to control it increases, more and more mHealth devices appear on the market. Smartphones and smartwatches are the most popular, but that's not all. One of the most interesting proposals is Google Glass, a gadget worn on the head, which is equipped with an accelerometer, gyroscope, and a camera, thanks to which we can get to know our heart rate (HR) and respiratory rate (RR). Another noteworthy invention is Plaster Zio. It is a waterproof patch applied to the left chest that provides a single-lead ECG and is used for continuous heart rhythm monitoring. The patch can be worn for up to 14 days and provides relatively long-term heart rate monitoring without the need to replace the battery or recharge, and includes an event marker button that can be pressed when a patient experiences symptoms (9, 10). Another example is AliveCor's Kardia Mobile, an ECG event recorder for smartphones that provides 30 sec of ECG. It integrates ECG leads in a smartphone case and enables the recording of heart rhythms and then electronic sharing of the recordings (10). Currently, Kardia Mobile devices from AliveCor enable recording one or even six ECG leads. Recently, a device in the form of a card with the same functions has also been made available for general use. Another type of heart rate monitoring device is the chest belt. Examples of these devices include BioHarness and Polar devices, among others. In addition to monitoring the heart rhythm, they enable the monitoring of physical activity, respiratory rate and body temperature. These devices are dedicated primarily to people who practice sports intensively and want to monitor the circulatory and respiratory systems.

Current devices can monitor ECG, heart rate, arrhythmia, blood pressure, stress, respiratory rate, temperature, saturation, ischemia, and apnea. A comparison of the most popular devices used for mobile rhythm monitoring is presented in Table 1. Taking into account the capabilities of the existing mHealth devices, potential groups of patients for which the use of these devices would be an added value would be patients with palpitations, presyncope or syncope, heart failure, established AF, coronary artery disease, QTc prolongation in medical history, with a tendency to hyperkalemia, requiring cardiac rehabilitation and with peripheral vascular disease.

**Table 1:** Comparison of the most popular devices used for mobile rhythm monitoring.

**Device**	**Type of product**	**The method used to detect heart rate or rhythm**	**AF detection accuracies**	**Continuous HRV measurement**	**Other medical functionalities**	**FDA approval**
Apple Watch 4 or later (Apple, United States)	Watch	PPG—for long-term surveillance of HR Two-lead user-triggered ECG via one installed electrode in the digital crown and the other installed electrode in the back of the watch	90.5% (11)	No	PA, falls, sleep	Yes
KardiaMobile (AliveCor, United States)	Card, pocket mobile device	Up to six lead ECG	100% (11)	No	No	Yes
Fitbit Flex, One, Charge (Fitbit, United States)	Watch	PPG—an algorithm that can passively assess the heart rhythm in the background, and if there are any signs suggestive of AF, the user get notify	98.2% (12)	No	PA, sleep	Yes
Garmin (Garmin, United States)	Watch	PPG	nd	No	PA, sleep	No
Polar (Polar Electro, Finland)	Chest strap	Electrocardiac sensors which detects and monitor HR	nd	Yes	PA	No
Zio Patch (iRhythm Technologies, United States)	Patch	Single-use, adhesive, external ambulatory ECG device which continuously records up to 14 days of ECG data. The device has no external wires or electrodes; it records ECG data via a single lead contained in the device housing	100% (13)	No	No	Yes
Microsoft Band (Microsoft)	Band	PPG	nd	No	PA, sleep	No
BioHarness (Zephyr)	Chest belt	Continuous ECG sampled at 250 Hz by means of a couple of textrodes embedded on a chest strap	nd	Yes	PA, RR, skin temperature	Yes

AF, atrial fibrillation; ECG, electrocardiogram; HR, heart rate; HRV, heart rate variability; nd, no data; PA, physical activity; PPG, photoplethysmography; RR, respiratory rate.

Measurement of HR during exercise and rest can be used to predict the risk of cardiovascular disease. In healthy populations, high resting HR is associated with an increased risk of coronary artery disease and is an unfavorable predictor of outcomes in patients with established heart failure (14). Disturbed return to resting heart rate after exercise correlates with increased rate of adverse cardiovascular events. HR variability (HRV) is also strongly associated with the risk of adverse cardiovascular events in healthy subjects and heart failure patients with reduced ejection fraction (14).

A prolonged QT interval can predispose patients to life-threatening arrhythmias. The single-lead ECG patch BodyGuardian (Preventice Solutions Group, USA) and the algorithm-predicted QTc from the KardiaMobile 6-lead ECG compared to the 12-lead ECG confirmed the satisfactory quality of this type of measurements (15, 16). Hyperkalemia is another a reversible cause of life-threatening arrhythmias, which can manifest on the ECG in the form of: peaked T waves, QRS widening, PR interval shortening and bradycardia. It is observed especially in patients with chronic kidney disease. In these patients, efforts were made to evaluate the effectiveness of the detection of hyperkalemia with AliveCor devices. The sensitivity by the duration of hyperkalaemia was 94% and the specificity was 74%, however high false-positive, and false-negative rates and the need for continuous ECG monitoring decreased the enthusiasm supporting the use of wearables to find out hyperkalaemia (17). However, in the future, after improving the algorithms responsible for the detection of hyperkalaemia, it may be an interesting alternative to laboratory diagnostics in specific groups of patients. Cardiovascular telerehabilitation programs supported by real-time wearable data can revolutionize home-based rehabilitation and relieve the inconvenience and decrease the cost associated with center-based programs. A randomized trial comparing REMOTE-CR—a real-time, remote telerehabilitation platform that includes the use of a chest-worn wearable sensor (BioHarness 3, Zephyr Technology, United States) - with a classic center-based program showed that REMOTE-CR was associated with less sedentary time at 24 weeks and was even more cost-effective (18). In patients with peripheral vascular disease, the first-line treatment for intermittent claudication is a supervised exercise program of gradually increasing intensity. Two randomized trials with patients with peripheral vascular disease, each utilizing the ankle-based accelerometer StepWatch 3 (Modus, United States), proved that wearable-guided training was associated with a significant improvement in walking ability, speed, and peak oxygen consumption (19, 20).

Mobile applications for patients are very numerous and popular, which makes our phone a personal caregiver nowadays. Among the cardiological applications, the most popular are heart rate monitoring systems that simultaneously create history and graphs of the pulse wave. Some of them are related to algorithms for selecting/classifying ECG or other measurable symptoms of heart disease. In addition, you can also find programs for cardiac rehabilitation, blood pressure measurement, and systems for detecting heart failure. Other categories with a large number of cardiac applications include ECG education and interpretation, cardiology journals, calculators, and cardiopulmonary resuscitation (CPR) instructions (21).

Considering the potential of mHealth devices, the most important thing now would be to design a unified (at the beginning even within a given country) application that would enable direct data transfer to a doctor, for instance, when the patient's state of well-being has deteriorated.

Mobile devices have been conquering the world for several years. Recognizing their potential, the companies producing them try to meet customer requirements and the possibility of using them, for example in medicine. Despite the excellent results of research in this area, there are many problems that need to be solved in order to take advantage of their potential on a large scale. Barriers include heterogeneity in an application, lack of reimbursement structures, and socio-economic and demographic disproportions in access to technology. It is obvious that the devices mentioned above are most often used by young people who care about their health. Low-income people and older people will be less likely to access these tools, even though they are the group most likely to use them. When a patient uses mHealth devices, despite the diagnosis of arrhythmia by the application, there is no telemedicine network sufficiently built to send the results, such as ECG, to the doctor for evaluation. Some patients ignore the reports as irrelevant or are afraid of the diagnosis and initiation of chronic treatment (22). On the other hand, some patients may focus too much on interpreting the constantly accumulated information. The abundance of data can also, in extreme cases, become the basis for the overdiagnosis of diseases.

## Conclusions

It usually takes months to register arrhythmias on the basis of multiple ECG recordings and frequent admissions to emergency departments (23). More importantly, this period is usually very stressful both for pregnant patients, as in the presented case, and for the other patient awaiting diagnosis. Due to the growing popularity of mHealth technology in the general population, cardiologists gain access to more data, which will enable faster diagnosis and treatment of arrhythmias (24). Arrhythmia recording with Apple Watch and other ECG recorders can be of great diagnostic value in preventing complications of arrhythmia, such as ischemic stroke in the case of patients with AF. It is also an important supplement to diagnostics and enables faster diagnosis in patients with paroxysmal palpitations. The widespread use of devices connected to the Internet makes it possible to collect a large amount of clinical information, without the need to visit the doctor directly, at relatively lower costs for hospitals and clinics.

## Data availability statement

The original contributions presented in the study are included in the article/supplementary material, further inquiries can be directed to the corresponding author.

## Ethics statement

Written informed consent was obtained from the participant/s for the publication of this case report.

## Author contributions

MH, GS, and EL contributed to the conception and design of the study. MH wrote the first draft of the manuscript. MH, GS, EL, and DK wrote sections of the manuscript. All authors contributed to manuscript revision, read, and approved the submitted version.

## Conflict of interest

The authors declare that the research was conducted in the absence of any commercial or financial relationships that could be construed as a potential conflict of interest.

## Publisher's note

All claims expressed in this article are solely those of the authors and do not necessarily represent those of their affiliated organizations, or those of the publisher, the editors and the reviewers. Any product that may be evaluated in this article, or claim that may be made by its manufacturer, is not guaranteed or endorsed by the publisher.
